# Impulsive and premeditated aggression in male offenders with antisocial personality disorder

**DOI:** 10.1371/journal.pone.0229876

**Published:** 2020-03-06

**Authors:** Jacinto Azevedo, Maria Vieira-Coelho, Miguel Castelo-Branco, Rui Coelho, Margarida Figueiredo-Braga

**Affiliations:** 1 Department of Neuroscience and Mental Health, Faculty of Medicine, University of Porto, Porto, Portugal; 2 i3S – Institute for Research and Innovation in Health, University of Porto, Porto, Portugal; 3 Department of Biomedicine – Therapeutics and Pharmacology Unit, Faculty of Medicine, University of Porto, Porto, Portugal; 4 CiBit - Coimbra Institute for Biomedical Imaging and Translational Research, ICNAS, University of Coimbra, Coimbra, Portugal; Xi’an Jiaotong University School of Medicine, CHINA

## Abstract

**Introduction:**

Aggression is a clinical symptom of various psychiatric disorders that can be conceptualised as a physical act towards another person with the intent to cause harm. In antisocial personality disorder (ASPD), aggression is a frequent manifestation that differently compromise therapeutic and prognostic goals according to its impulsive or premeditated categorisation. ASPD is characterised by high levels of impulsivity, psychopathic traits, and a high prevalence of co-morbid substance use disorders (SUDs). Aggression in ASPD patients may determine long and recurrent imprisonment thus representing a challenge clinicians and legal experts face.

**Objectives:**

Our aims were to characterise impulsive and premeditated aggression in male ASPD offenders as well as to determine the potential role of SUDs, impulsivity, and psychopathic traits as predictors.

**Materials and methods:**

In this cross-sectional study we evaluated a sample of ASPD offenders with a battery of clinical and psychometric, standardised instruments: the Psychopathy Checklist-Revised (PCL-R), the European Version of the Addiction Severity Index (EuropASI), the Barratt Impulsivity Scale Version 11 (BIS-11), and the Impulsive/Premeditated Aggression Scale (IPAS).

**Results:**

We used a total sample of 134 offenders, all of whom were male. ASPD patients (n = 96) had a 71.9% prevalence of impulsive aggression and a 28.1% prevalence of premeditated aggression. ASPD patients with impulsive aggression had significantly lower scores of total PCL-R (p<0.01) factor 1 and interpersonal facet 1 (p<0.05), compared with ASPD patients with premeditated aggression. ASPD patients with impulsive aggression and ASPD patients with premeditated aggression had comparable BIS-11 mean scores, and exhibited an equal prevalence of SUDs. The interpersonal facet 1of the PCL-R predicted the aggression type (p<0.05) in ASPD patients, and the exponential beta value for facet 1 was 1.42 (CI = 1.03; 1.95).

**Conclusions:**

The aggression type that is associated with ASPD is mainly impulsive in nature. ASPD patients who have higher scores of psychopathic traits have a lower probability of exhibiting impulsive aggression and a higher probability of exhibiting premeditated aggression. Although ASPD patients have high levels of impulsivity and a high frequency of SUDs, these two variables were not predictors of the aggression type.

## Introduction

Physical aggression is common in individuals with antisocial personality disorder (ASPD) and is linked to criminal acts, psychopathy, impulsivity, and substance use disorders (SUDs) [1]. Although aggression expression is important on the individual psychological level, being able to adequately inhibit aggression is a healthy personality characteristic [2].

Research on aggressive behaviours in incarcerated populations is supported by the World Health Organization due to its double interest: it is an issue of a minority population, and it is related to high levels of human suffering. Aggression is a public health issue that can certainly be prevented [3].

The prison context is characterised by a high prevalence of aggressive acts and inmate-to-inmate victimisation [4]. The way aggression is inhibited and prevented in prison involves judicial and medical strategies through a biopsychosocial model [5]. The prison population has a high prevalence of mental pathology that can be related to aggressive acts, and the most common mental disorders in prisons include ASPD, SUDs, depressive disorders, and anxiety disorders [6, 7].

The assessment of aggressive acts involves the evaluation of an individual’s physical and mental health state as well as the motivations that led to those aggressive acts [8]. When describing an aggressive act, we should characterise the individual’s level of planning and possible understanding of hypothetical consequences, the presence of frustrations, insults, interpersonal attack, threats, environmental stressors, and the presence of psychiatric disorders [9].

Human aggression can be classified into two types: impulsive aggression, also called reactive aggression, and premeditated aggression, also called instrumental or proactive. Non-dichotomic classifications include other forms of aggression namely those associated with psychotic psychopathology [10]. This classification of aggression allows the characterisation of individuals according to sociodemographic, criminal, neurophysiological, and clinical variables [11].

Acts of impulsive aggression are characterised by uncontrolled and exaggerated responses to the stimuli which provoke them. Individuals who exhibit this type of aggression tend to show high levels of physiological arousal associated with stress, neurocognitive difficulties, and impulsive personality traits [12, 13].

Conversely premeditated aggression corresponds to acts that materialise a previously defined plan aiming at a specific kind of gain. This type of aggression is associated with individuals who not only exhibit low physiological activation associated to stress, but who also present psychopathic personality traits [12, 13].

The clinical approach to aggression implies that each aggressive act should be classified as impulsive or premeditated such that the best possible strategy is selected at each moment [5, 14]. A fundamental clinical benefit of categorising aggressive acts is to determine the utility of pharmacological treatments for aggression. In particular, and contrary to individuals who display premeditated aggression, those who demonstrate impulsive aggression tend to benefit from the currently available pharmacological treatments [8]. Clinicians should be aware of the fact that the same individual may present acts of premeditated or impulsive aggression throughout their life.

For clinical and research purposes, aggression can be categorised using self-administered instruments, such as the Impulsive/Premeditated Aggression Scale (IPAS) [15] and the Reactive-Proactive Aggression Questionnaire (RPQ) [16, 17]. Some authors, however, alert that the concepts of impulsive and premeditated aggression measured by the IPAS do not totally overlap with the concepts of proactive or reactive aggression measured by the RPQ and should therefore not be used interchangeably. It has been suggested that the RPQ focuses more closely on actual aggressive behaviour, while the IPAS focuses more closely on emotions and their regulation [18, 19].

It is important to distinguish the concept of aggression from the concept of impulsivity and, even more, impulsive aggression from impulsivity. The clinical conceptualisation of impulsivity, according to the Diagnostic and Statistical Manual of Mental Disorders, Fifth Edition (DSM-V), implies a behavioural and cognitive disinhibition, an immediate reaction to stimuli, an unplanned reaction at the spur of the moment, or a reaction with no regard for consequences [20].

The Barratt Impulsiveness Scale (BIS-11) is one of the most widely used psychometric instruments for research purposes concerning impulsivity [21]. The definition of impulsivity easily leads to the intuitive relationship between impulsivity and impulsive aggression; although they are related concepts, impulsivity seems to be present in any type of aggressive act and does not make a distinction between acts of premeditated or impulsive aggression [17].

The relationship between impulsive aggression, premeditated aggression, and impulsivity is highlighted in various studies that address the classification of aggression.

For example, impulsivity—measured by the BIS-11—was positively correlated with both impulsive and premeditated aggression dimensions as measured by the IPAS [15, 22, 23] as well as proactive and reactive aggression dimensions as measured by the RPQ [24, 25]. Thus, when we refer to impulsive individuals, we may be referring to aggressive individuals without knowing what kind of aggression they will manifest. Because impulsivity does not allow the categorisation of aggression, there was a need to develop reliable and specific instruments to measure impulsive and premeditated aggression types [17].

ASPD is a psychiatric diagnosis defined as a pervasive and persistent pattern of behaviour and emotional response characterised by a failure to conform to lawful and ethical behaviour; an egocentric, callous lack of concern for others, accompanied by deceitfulness, irresponsibility, manipulativeness, risk taking, and high levels of impulsivity. To be diagnosed with ASPD, an individual must be aged 18 years or older at the time of diagnosis and should have displayed features of conduct disorder prior to the age of 15 [20].

One of the most relevant features of ASPD is its high prevalence, which is 3% in the general population and may reach up to 50% in the prison population [6, 26–28].

Aggressive behaviour is a symptom of ASPD that is associated with poor prognosis and difficult treatment. ASPD is the only psychiatric disorder associated with an increase of impulsive and premeditated aggression acts [29–31].

On the other hand, children and adolescents who display aggressive acts are more prone to developing ASPD in their adult lives [32], which reveals the importance of an early therapeutic intervention for aggressive children and adolescents. Very little research has investigated the treatments which might be useful in aggressive ASPD patients [26].

Psychopathy is a concept related to ASPD, and discussion has been held regarding the relationship between the two. ASPD tends to focus on antisocial behaviour, while psychopathy tends to focus on emotional dysregulation [29]. There seems to be some consensus that psychopathy corresponds to ASPD individuals with severe emotional dysregulation.

Psychopathy can be defined as a clinical syndrome composed of a set of characteristics that manifest in interpersonal relationships, affective reactions, and behaviours. Affected individuals demonstrate egocentrism, pathological lying, manipulation, irresponsibility, impulsivity, novelty pursuit, limited behavioural control, insensitive affection, lack of empathy, guilt, or remorse, and a set of unethical and antisocial behaviours that are not necessarily criminal. These characteristics appear to be hereditary, manifest in childhood, and are relatively stable throughout adolescence and adulthood [33].

The Psychopathy Checklist-Revised (PCL-R) is the gold standard for the diagnosis of psychopathy [28]. Scientific evidence proves that the concept of psychopathy allows us to define a group with distinct biological, physiological, and psychological characteristics [34]. Moreover, as a model, psychopathy is useful for studying empathy, social behaviours such as avoidance of learning, social cooperation, emotional processing, and moral behaviours [35]. Psychopathy is a strong predictor of both criminal recidivism and premeditated aggression [36, 37].

SUDs are highly comorbid with ASPD and are related to both aggression and criminal behaviour [38, 39]. Although they represent two different nosological entities, some authors argue that SUDs may be part of the spectrum of antisocial behaviour [40]. Individuals with an SUD possess increased levels of impulsivity, which may be explained by the impact of abuse substances on the brain’s structure and function as well as by previous individual vulnerability [41]. Aggression in these individuals can be of the impulsive or premeditated aggression type [42, 43].

The present study’s objective was to firstly determine the prevalence of impulsive and premeditated aggression in patients with ASPD in a prison context, and secondly determine whether or not impulsivity, psychopathy, and SUD are predictors of aggression type.

## Material and methods

### Population

The sample of the present study included 134 male inmates of a medium-high security penitentiary institution in the North of Portugal. The subjects were recruited through the use of a convenience sampling strategy between January and March of 2015. All the individuals in this institution had received sentences longer than 10 years. The penitentiary institution had a total number of 710 inmates at the time of the protocol application. The research protocol was formally approved by the ethics committee of the *Centro Hospitalar de São João* (Document no. 48.14) and the hosting institution, the General Direction of Probation and Prison Services. Participation was voluntary, no reward was offered in exchange for participation. The participants were able to leave the research at any time without any consequences, and the individuals who decided not to participate received the same treatment offered to participants. In accordance with the Declaration of Helsinki, written, informed consent was obtained after explaining the procedures to each participant. The study followed a cross-sectional design.

Participants were included if they were aged 18 years or older and had been referred to the clinical services after preforming acts of physical aggression towards other inmates. Their ability to read and provide their written, informed consent was also considered for their inclusion. Participants were excluded if they exhibited a psychiatric or neurological disorder in an acute and non-treated episode.

### Procedures and instruments

**Fig 1:**
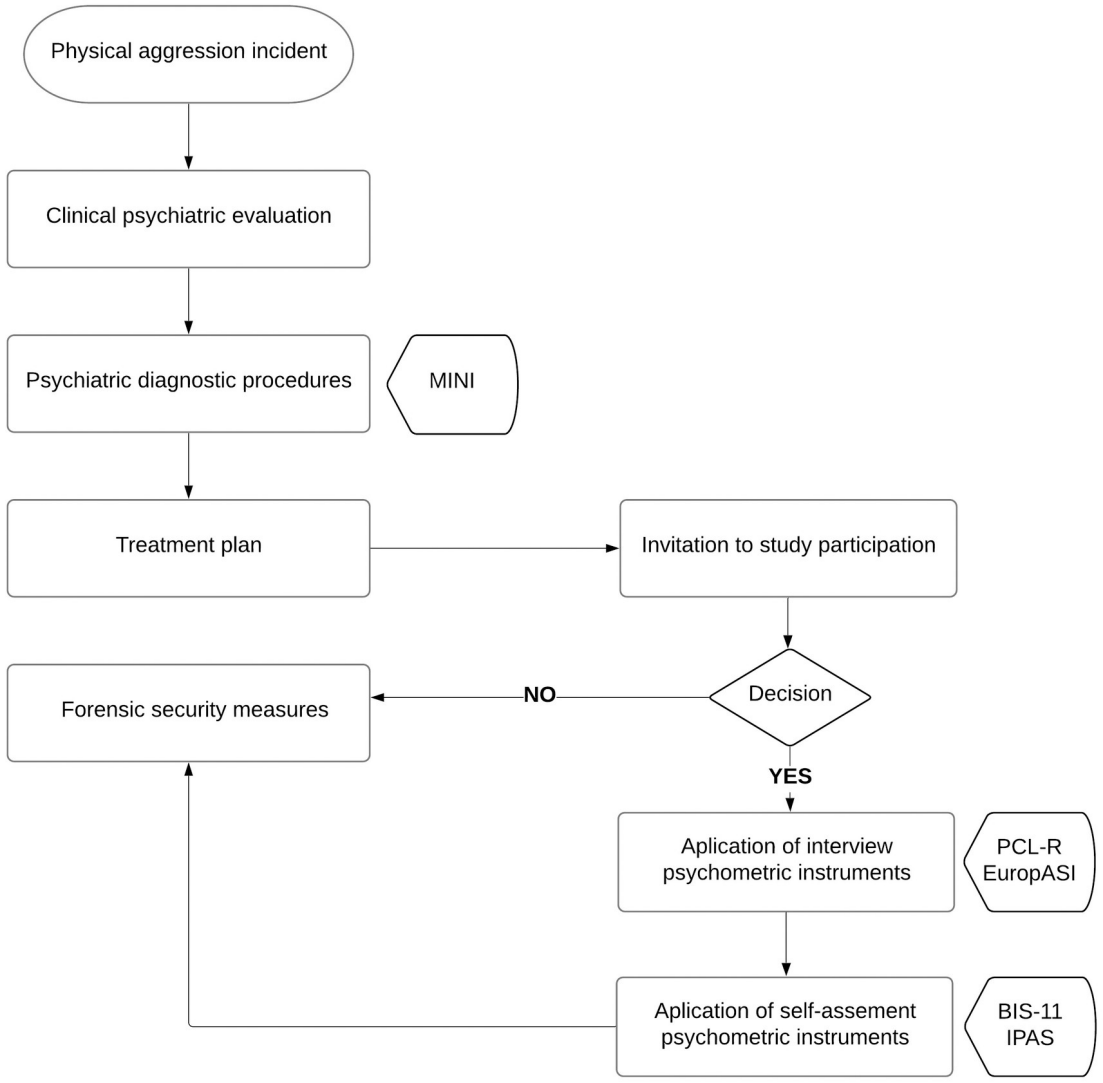
Study protocol flow chart. MINI—Mini-International Neuropsychiatric Interview, PCL-R–Psychopathy checklist revised, EuropASI—Addiction severity index European version, BIS-11—Barratt Impulsivity Scale 11, IPAS—Impulsive Premeditated Aggression Scale.

After the occurrence of a physical aggression incident in the forensic institution the inmates were referred for a clinical psychiatric evaluation. The local forensic psychiatrist, who had prior clinical knowledge of all the institution inmates, established the psychiatric diagnoses using the Mini-International Neuropsychiatric Interview (MINI). After the establishment of the appropriated treatment plan for each individual, the participation in the study protocol was proposed to the inmates. Those who agreed to participate after verbal information about the study protocol and procedures signed an informed written consent form and were submitted to the application of four psychometric instruments: two in the form of a standardized clinical interview, the Psychopathy checklist revised (PCL-R) and the Addiction severity index European version (EuropASI); and two self-administration psychometric instruments, the Barratt Impulsivity Scale eleven version (BIS-11), and the Impulsive Premeditated Aggression Scale (IPAS). The participants were interviewed and submitted to the psychometric assessment in the penitentiary institution’s clinical department. Finally, all inmates were subject to forensic security measures and received the appropriate treatment whether or not they had accepted to participate in the study protocol (Fig 1).

The primary outcome was the categorisation of aggression by applying IPAS, while the secondary outcomes were impulsivity, psychopathic traits, and the presence and severity of SUDs.

## Mini-International Neuropsychiatric Interview

The MINI is a short, structured, diagnostic interview for psychiatric disorders and is performed according to the DSM-IV and ICD-10 criteria. It was designed to allow a brief and accurate psychiatric evaluation, which proves useful in clinical trials and epidemiologic studies. In this study, the MINI was used to diagnose ASPD and psychiatric co-morbidities, such as SUDs, as well as anxiety and depressive disorders. Participants were assessed with the Brazilian Portuguese version 5.0.0 of the MINI [44].

The specificity values for the diagnoses performed with the MINI’s aid were between 0.95 and 0.97, while the sensitivity values were between 0.67 and 0.94 [45].

### Psychopathy checklist-revised

The PCL-R measures psychopathic traits by collecting information from clinical records and conducting a semi-structured interview [28]. The 20 items that compose the PCL-R are scored as ‘absent’ (0), ‘present to some degree’ (1), or ‘fully present’ (2), thus allowing a maximum total score of 40 points. A score of 30 was set as the cut-off value for psychopathy. The PCLR’s structural analysis defends that it can be interpreted as a four-factor model that comprises interpersonal, affective, lifestyle, and antisocial facets. The interpersonal and affective facets jointly represent the core traits of the psychopathic personality and constitute the second-order factor ‘1’, which is related to emotional dysfunction, while the lifestyle and antisocial facets form the second-order factor ‘2’, which is related to antisocial behaviour [33].

The PCL-R’s reliability was recently re-evaluated (Cronbach’s alpha value: total score = 0.87, factor 1 = 0.86, facet 1 = 0.77, facet 2 = 0.79, factor 2 = 0.86, facet 3 = 0.79, facet 4 = 0.79) [46]. The PCL-R’s structural properties had been validated in Portuguese-speaking samples [47] with a kappa index of 0.87 and a sensitivity measure of 84.8% [48].

The PCL-R’s quotation implies extensive clinical knowledge of the patients was obtained during the interview as well as from alternative sources. In this study, the PCL-R was quoted by the admitted patients’ assistant psychiatrist. This psychiatrist (with eight years of experience working with ASPD patients in forensic settings) had access to each patient’s clinical information as well as information from other sources, namely records from other physicians who had contact with the patient, legal and court records, and prison records held by guards and educators; in other words, the psychiatrist that quoted the PCL-R had detailed clinical and contextual knowledge of each participant.

### European version of the Addiction Severity Index

The EuropASI was applied to access the SUDs’ severity. This semi-structured interview offers an inventory of problems that occurred over the previous month in six areas: physical health, work income, drug use, legal status, family and social relationships, and psycho-emotional status. The EuropASI also assesses one’s history of suicide attempts, and one’s criminality type. This multidimensional clinical and research instrument is an adapted version of the Addiction Severity Index (fifth version) [49].

The composite scores of each dimension ranged from 0 to 1, while higher scores indicated greater severity. The reliability measures indicated moderate to good internal consistency in the European samples (Cronbach’s alpha: 0.69–0.92) [50, 51].

### Barratt Impulsivity Scale Version 11

The Barratt Impulsivity Scale Version 11 (BIS-11) is a self-report questionnaire used to assess a patient’s general impulsivity [52]. The current scale version comprises 30 items that are rated from 1 (rarely/never) to 4 (almost always/always). Factor analyses revealed six first-order factors (attention, cognitive instability, motor, perseverance, self-control, and cognitive complexity) and three second-order factors (attentional, motor, and non-planning). The BIS-11’s structural properties were replicated in Portuguese-speaking subjects [53]. The Portuguese version has recently been re-evaluated, and the following Cronbach’s alpha values were reported for each score: BIS-11 total score = 0.84, attention dimension = 0.80, cognitive instability dimension = 0.62, motor dimension = 0.84, perseverance dimension = 0.53, self-control dimension = 0.80, and cognitive complexity dimension = 0.67 [54].

### Impulsive/Premeditated Aggression Scale

The IPAS is a 30-item self-report questionnaire used to rate aggressive acts that occurred over the previous six months [15]. Items are scored on a five-point Likert scale ranging from 1 (strongly disagree) to 5 (strongly agree). The scale differentiates two factors—premeditated aggression, here referred to as ‘PM’ and impulsive aggression, here referred to as ‘IA’—that can be scored either dimensionally or categorically (Stanford MS, Classification procedures, unpublished manual). Discrete categories (impulsive vs premeditated) are obtained by a categorical approach in which only the percentage of the positive items (5 = strongly agree or 4 = agree) for each aggression scale is calculated (Stanford MS, Classification procedures, unpublished manual). For the IA scale, sensitivity was 0.96 and specificity was 0.50. For the PM scale, sensitivity was 0.60 and specificity was 0.96 [15]. All IPAS validation studies report identical results through a principal component analysis with the two factors of IA and PM. Internal consistency coefficients varied between 0.70 and 0.93 for IA and between 0.66 and 0.90 for PM.

The structural properties of the IPAS were previously validated in a Portuguese forensic population. Each individual’s IA and PM levels were obtained through the sum of 21 of the IPAS’s 30 items; the IA factor comprised ten items (30, 27, 22, 9, 24, 15, 26, 4, 7, and 13), while the PM factor comprised eleven items (6, 14, 29, 28, 2, 23, 12, 16, 20, 10, and 1). In the Portuguese validation study, inmates’ IA and PM subscales scored Cronbach’s alpha values of 0.89 and 0.88, respectively [23].

### Statistical analyses

Analyses were carried out using IBM SPSS Statistics for Mac, Version 22.0 (Armonk, NY, USA: IBM Corp.) Descriptive statistical measures were used to characterise the groups. Parametric (t-test for independent samples) and/or non-parametric tests (e.g., Chi-square and Mann-Whitney tests) were used to test the hypotheses, and a logistic regression analysis was employed to investigate the significant variables associated with the prediction of aggression categorisation using Vittinghoff’s recommendations for small samples [55].

## Results

### Sociodemographic, criminal and clinical characterisation

The sample comprised 134 individuals with a mean age of 37.8 ± 9.2 years, of which 71.9% (n = 96) were single and 49.4% (n = 66) had children. The mean education level was 7.1 ± 2.9 years, while the mean length of imprisonment at the time of assessment was 112.9 ± 70.6 months. A total of 64.2% (n = 86) of the inmates had been convicted for violent crimes (physical assault, murder, or attempted murder).

In the entire sample, 71.6% (n = 96) of the individuals had ASPD, 56.7% (n = 76) of the individuals presented SUDs, 31.5% (n = 42) had depressive disorders, 28.1% (n = 37) had anxiety disorders, 28.4% (n = 38) exhibited psychopathy, 30.3% (n = 41) had personal histories of suicide attempts (Table 1).

**Table 1:** Sociodemographic, criminal and clinical characterisation according to the presence of ASPD.

	Without ASPD n = 38	With ASPD n = 96	p-value
**Sociodemographic characterisation**			
Age (years)^1^	38.13 (8.02)	37.62 (9.70)	0.71^a^
Education (years)^1^	7.13 (2.79)	7.12 (3.06)	0.12^a^
Children ^2^	21 (54.8)	45 (46.6)	0.48^b^
Single^2^	28 (74.2)	68 (70.7)	0.60^b^
**Criminal characterisation**			
Time spent in prison^1^ (months)	107.70 (68.79)	115.67 (72.0)	0.43^a^
Violent crimes^2^	19 (50)	67 (69.8)	0.03^b^
**Psychiatric characterisation**			
Substance use disorders^2^	15 (39.5)	61 (63.5)	0.01^b^
EuropASI^2^	1.56 (1.03)	1.54 (1.26)	0.64^a^
Active drug use^2^	7 (19.4)	40 (41.6)	0.06 ^b^
SUD in treatment^2^	15 (39.5)	24 (25.0)	0.16 ^b^
Depressive disorders^2^	16 (41.9)	25 (25.9)	0.74^b^
Anxiety disorders^2^	12 (32.3)	25 (25.9)	0.51^b^
Psychopathy^2^	na	38 (39.2)	
Suicide attempts^2^	9 (23.6)	33 (34.5)	0.53 ^b^

ASPD–Antisocial personality disorder, SUD–Substance use disorder, EuropASI–European version of the Addiction Severity Index

^1^ Mean (SD).

^2^ n (%).

^a^ Mann-Whitney Test.

^b^ Chi-square Test.

na–not applicable.

### Comparison between individuals according to the presence of antisocial personality disorder

Impulsive aggression was detected in 71.8% (n = 69) of the individuals with ASPD, while premeditated aggression was detected in 28.2% (n = 27) of the individuals with ASPD. Individuals with ASPD had a lower frequency of impulsive aggressive acts (p<0.01, OD = 0.22, CI = 0.06, 0.77) and a higher frequency of premeditated aggressive acts (p<0.05, OD = 3.87, CI = 1.04, 14.47) than individuals without ASPD.

Individuals with ASPD had a higher mean score in the IPAS premeditated aggression dimension (p<0.05) than individuals without ASPD. Individuals with ASPD and those without ASPD had comparable scores in the IPAS impulsive aggression dimension.

Individuals with ASPD and those without ASPD were comparable in terms of their sociodemographic characteristics, their prevalence of anxiety and depressive disorders, and their personal history of suicide attempts.

Individuals with ASPD had a higher mean score in all PCL-R factors and facets (p<0.05) than individuals without ASPD. The BIS-11 impulsivity measure was comparable in both groups—those with and without ASPD (Table 2).

**Table 2:** IPAS, PCL-R and BIS-11 according to the presence of ASPD.

	Without ASPD n = 38	With ASPD n = 96	p-value
**IPAS**			
Impulsive aggression dimension^1^	31.48 (9.23)	29.55 (8.28)	0.22 ^a^
Premeditated aggression dimension^1^	22.5 (9.31)	28.05 (8.54)	0.05 ^a^
Aggression—Impulsive type^2^	35 (92.1)	69 (71.9)	0.01 ^b^
Aggression—Premeditated type^2^	3 (7.9)	27 (28.1)	0.01 ^b^
**PCL-R total**^1^	16.92 (7.62)	26.71 (6.99)	0.01^c^
Factor 1 ^1^	5.16 (4.31)	11.0(4.56)	0.01 ^b^
Factor 2 ^1^	11.23 (5.17)	15.21 (4.24)	0.01 ^b^
F1-Interpersonal ^1^	2.58 (2.75)	5.76 (2.44)	0.01 ^b^
F2-Affective^1^	2.58 (2.05)	5.24 (2.91)	0.01 ^b^
F3-Lifestyle^1^	7.77 (2.85)	8.45 (2.33)	0.01 ^b^
F4-Antisocial^1^	4.45 (2.74)	6.76 (2.52)	0.01 ^b^
**BIS-11 total**^1^	54.58 (21.67)	58.24 (23.58)	0.24 ^b^
Attentional^2nd^ ^1^	14.35 (6.35)	14.10 (6.60)	0.88 ^b^
Motor^2nd^ ^1^	19.06 (8.16)	18.93 (9.06)	0.63 ^b^
Nonplanning^2nd^ ^1^	21.16 (9.16)	22.10 (10.39)	0.53 ^b^
Attention^1st^ ^1^	9.00(4.17)	8.16 (4.02)	0.36 ^b^
Cognitive instability^1st^ ^1^	5.35 (2.47)	5.95 (3.05)	0.12^b^
Motor^1st^ ^1^	12.39 (5.99)	11.98 (6.51)	0.12 ^b^
Perseverance^1st^ ^1^	6.68 (3.16)	6.95 (3.40)	0.78 ^b^
Self-control^1st^ ^1^	10.90 (5.71)	11.38 (5.85)	0.63 ^b^
Cognitive complexity^1st^ ^1^	10.26 (4.29)	10.72 (4.97)	0.38 ^b^

IPAS–Impulsive Premeditated Aggression Scale, PCL-R–Psychopathy Checklist Revised, BIS-11 –Barratt Impulsivity Scale 11, ASPD–Antisocial personality disorder

^1^ Mean (Standard deviation).

^2^ n (%),

^a^ Mann-Whitney Test.

^b^ Chi-square Test.

^c^ Independent samples T-Test.

By comparing ASPD individuals with psychopathy (n = 37) to ASPD individuals without psychopathy (n = 59), we observed that, in our sample, they were comparable in terms of sociodemographic characteristics, psychiatric co-morbidities, as well as BIS-11 and EuropASI scores.

ASPD individuals with SUDs had higher mean scores in the self-control first-order factor and non-planning second-order factor of the BIS-11 than ASPD individuals without SUDs (p<0.05).

Individuals with ASPD had a higher frequency of both violent crimes (p<0.05, OD = 2.3, CI = 1.1, 5.0) and SUDs (p<0.05, OD = 2.67, CI = 1.24, 5.78) than individuals without ASPD. Addiction severity in all the EuropASI dimensions was similar in both groups either with or without ASPD (Table 1).

### Comparison of individuals with antisocial personality disorder according to impulsive and premeditated aggression

ASPD individuals with impulsive aggression (n = 69) had significantly lower scores of total PCL-R (p<0.001; CI = 4.38, 9.49), factor 1, and facet 1 (interpersonal) (p<0.05) than ASPD individuals with premeditated aggression (n = 27). ASPD individuals with impulsive aggression had a lower probability of exhibiting psychopathy (OD = 0.19, p = 0.001, CI = 0.07, 0.50) than those with premeditated aggression. ASPD individuals with premeditated aggression had a higher probability of showing psychopathy (OD = 5.26, p = 0.001, CI = 2.02, 13.70) than those with impulsive aggression, while ASPD individuals with psychopathy had a 51.4% prevalence of impulsive aggression (n = 19).

ASPD individuals with impulsive aggression were similar to ASPD individuals with premeditated aggression regarding impulsivity, as measured by the BIS-11, in both the total score and the first and second-order factors. ASPD individuals with impulsive aggression were similar to ASPD individuals regarding premeditated aggression in their sociodemographic and criminal characteristics, prevalence and severity of SUDs, prevalence of anxiety and depressive disorders.

### Aggression type predictors in individuals with antisocial personality disorder

A binary logistic regression was performed to determine possible predictive factors of aggression type in ASPD individuals. Using a forward, stepwise, conditional methodology, variables that were different between impulsive and premeditated ASPD individuals were included in the analysis, including psychopathy, the PCL-R, factor 1, and facet 1 (interpersonal). Facet 1 reliably distinguished between impulsive and premeditated aggression in ASPD individuals (Chi-square = 6.1, p<0.05, with df = 1, Nagelkerke’s R^2^ of 0.14). The exponential beta value for facet 1 was 1.42 (CI = 1.03, 1.95), meaning that, for each increase in one unit of the interpersonal facet, the OD for premeditated aggression increased by 1.42.

### Comparison of individuals according to the presence of violent crimes

Individuals who had committed violent crimes (n = 86) had higher mean scores of PCL-R factor 1 and facet 2 than those who had not committed violent crimes (n = 48) (p<0.05). The frequency of violent crimes was higher in individuals with ASPD (p<0.05, OD = 2.31, CI = 1.07, 4.99), lower in individuals with SUDs (p<0.01, OD = 0.34, CI = 0.16, 0.73), and higher in single-status individuals (p<0.05, OD = 2.91, CI = 1.07, 7.94) than the frequency of individuals who did not commit violent crimes. Individuals who committed violent crimes and those who did not commit violent crimes were comparable in their aggression categorisation, BIS-11 scores, EuropASI scores, prevalence of anxiety and depressive disorders, and in their personal history of suicide attempts.

### Impulsive and premeditated aggression dimension correlations with BSI-11, PCL-R and EuropASI

Impulsive aggression, expressed as a dimension, was positively correlated with the BIS-11 total score and all first- and second-order factors (p<0.05). Impulsive aggression wasn´t correlated with neither the PCL-R nor the EuropASI.

Premeditated aggression, expressed as a dimension, was positively correlated with the BIS-11 total score and all first- and second-order factors (p<0.05). Premeditated aggression was positively correlated with the PCL-R total score, facet 1 (interpersonal), and facet 3 (lifestyle) (p<0.05). Premeditated aggression was not correlated with the EuropASI (Table 3).

**Table 3:** Correlations between impulsive and premeditated aggression dimensions of IPAS with BIS-11, PCL-R and EuropASI.

	Impulsive Aggression n = 134	Premeditated Aggression n = 134
BIS-11	0.584*	0.477*
Attentional^2nd^	0.542*	0.542*
Motor^2nd^	0.484*	0.601*
Nonplanning^2nd^	0.642*	0.383*
Attention^1st^	0.362*	0.425*
Cognitive instability^1st^	0.409*	0.613*
Motor^1st^	0.478*	0.652*
Perseverance^1st^	0.246*	0.265*
Self-control^1st^	0.291*	0.459*
Cognitive complexity^1st^	0.450*	0.485*
PCL-R	-0.027	0.221*
Factor 1	-0.005	0.22*
Factor 2	-0.044	0.131
F1-Interpersonal	-0.012	0.268*
F2-affective	-0.001	0.132
F3-Lifestyle	-0.024	0.170
F4-Antisocial	-0.052	0.070
EuropASI^1^	-0.025	-0.044
Medical^1^	-0.167	0.041
Economic^1^	-0.071	-0.017
Drug^1^	-0.007	-0.026
Legal^1^	-0.077	-0.129
Family^1^	0.011	0.188
Social^1^	0.087	-0.122
Psychiatric^1^	0.007	-0.042

IPAS–Impulsive Premeditated Aggression Scale, BIS-11 –Barratt Impulsivity Scale 11, PCL-R–Psychopathy Checklist Revised, EuropASI–European version of the Addiction Severity Index.

*Spearman’s rho correlation values significant at p<0.05.

## Discussion

In this study we observed that ASPD individuals commit less impulsive aggressive acts and more premeditated acts than individuals without ASPD. Nevertheless, impulsive aggression was the principal aggression type in ASPD individuals.

Furthermore, the interpersonal facet 1 of PCL-R was a predictor of the aggression type in ASPD patients. Specifically, individuals with ASPD who presented glibness, superficial charm, grandiose sense of self-worth, pathologic lying, conning, and manipulative behaviour (emotional insensitivity and dysfunction) were more likely to exhibit acts of premeditated aggression, while individuals with preserved emotional function were more likely to express impulsive aggression.

These personality characteristics emerged as predictors of premeditated aggression that point to the importance of emotional insensitivity when learning about how aggressive behaviour is used to obtain personal benefits [56, 57]. Conversely, others have stated that a high score in facet 1 can be considered a protective factor for impulsive aggression [58].

In this study, individuals with ASPD and psychopathy exhibited a higher prevalence of premeditated aggression than impulsive aggression. However, when considering the psychopathy diagnosis, the prevalence of premeditated and impulsive acts was similar, with a 1:1 ratio.

The effect of the psychopathic traits on the distinction between individuals with premeditated and impulsive aggression was also verified by the absence of correlations between the impulsive aggression dimension of the IPAS and the PCL-R in addition to the presence of a positive correlation between the premeditated aggression dimension and the total score of the PCL-R, facet 1 (interpersonal), and facet 3 (lifestyle) of the PCL-R.

Several authors suggest that the presence of psychopathy is related to an increase in an individual’s number of premeditated aggressive acts [30, 59–62]. Nevertheless, according to a recent meta-analysis, psychopathy is related in the same way to premeditated and impulsive aggression. [37]. This aspect is of clinical and legal relevance, as the relationship between psychopathy and premeditated aggression should be carefully examined by clinicians and lawyers regarding individuals with ASPD; moreover, psychopathy may exhibit impulsive and premeditated aggressive behaviours.

In the present sample, the scores obtained from the PCL-R as well as the prevalence of psychopathy were higher (meaning more dangerous individuals with severe personality disorders) than those reported by other authors in prison populations (roughly 15%) [28]. This may be explained by the fact that the sample was collected in a high-security prison.

### Sociodemographic and criminal characteristics according to aggression type

Aggression type was not associated with the studied sociodemographic or criminal characteristics of age, educational level, relationship status, presence of offspring, criminality type, or average time spent in prison, thus reinforcing the predictive value of facet 1. In fact, physical aggression towards others tends to arise in childhood and is maintained throughout life—regardless of the types of relationships maintained and offspring produced—and is additionally related to different types of crime [63].

This study’s participants demonstrated a mean age, educational level, relationship status, presence of offspring, criminality type, and average time spent in prison similar to those described for the Portuguese prison population with the same type of criminal record (General Direction of Probation and Prison Services, unpublished data).

In the entire sample, individuals with a history of conviction for violent crimes had a higher prevalence of ASPD, a lower prevalence of SUDs, and higher scores on the PCL-R, although the history of conviction for violent crimes had no impact on the classification of current aggressive acts. Violent crime has been associated with emotional insensitivity, and in our study, we replicate this finding [64].

### Co-morbid mental disorders with antisocial personality disorder and aggression type

In this study’s sample, the prevalence of SUD co-morbidity with ASPD was high and comparable to other studies. Other authors have also reported a high prevalence of this co-morbidity, at 55% [65], 53% [38], 84.5% [66], and 90% [67]. The prevalence of SUDs in individuals with ASPD is up to five times higher than that in individuals without ASPD [68]. A low prevalence of SUD co-morbidity with ASPD (40.6%) has also been reported [69], and the aggression type did not differ due to the presence or absence of an SUD. Current drug use and the number of individuals in treatment for SUDs were comparable across both groups.

We expected that individuals with SUDs would have a significant increase in their frequency of impulsive acts, although this result was not realised. We also verified that neither impulsive nor premeditated dimensions of aggression correlated with the SUD severity as measured by the EuropASI. Nevertheless, drug use and problems related to consumption, such as money debts, cravings, and withdrawal [70], may lead to an increase in impulsive aggression. It has been hypothesised that impulsive aggression and drug use may be part of the same spectrum of externalising behaviour [40, 71].

Interestingly, the presence of co-morbid depressive disorders had no effect on aggression type. Depressive disorders have been associated with poorer impulse control and aggressive behaviour, and patients with major depression have shown higher impulsivity and more severe aggression than controls and were more likely to commit violent crimes [72]. The occurrence of depression in this study’s ASPD patients was comparable to that reported by other authors, which varies from 10% to 54.2% [6, 66]. The aggressive behaviour in depressed patients may convey several manifestations, including suicidal attempts, whose prevalence in individuals with ASPD was comparable to that described in the literature and revealed no differences according to aggression type [73].

The prevalence of co-morbidity between ASPD and anxiety disorders reported in the literature is 50% [38, 66, 74]; a lower prevalence of anxiety disorders was observed in this study’s population, and no difference was found between the two aggression groups.

### Relationship between aggression type and impulsivity

Impulsivity, as measured by the BIS-11, was similar in individuals with and without ASPD. In ASPD patients, impulsivity was similar in individuals with impulsive aggression as well as in individuals with premeditated aggression, although individuals with premeditated aggression tended to have higher BIS-11 scores than those with impulsive aggression. The IPAS dimensions of impulsive and premeditated aggression had highly positive correlations with the BIS-11 total scores as well as first- and second-order factors. Previous studies report positive correlations between the BIS-11 and the IPAS impulsive aggression dimension as well as between the BIS-11 and the IPAS premeditated aggression dimension [22, 23]. Furthermore, impulsivity may be necessary to express aggression, but is not a predictive factor of aggression type [25].

### Limitations and future work

The small sample size and the fact that the sample exclusively comprised men may limit the results’ generalisation, as we do not know whether or not aggression in ASPD women exhibits similar characteristics. There are also limitations related to the probable influence of contextual variables, since research studies within a forensic facility context can change the way inmates respond to self-assessment instruments. Firstly, inmates may believe that their participation in research might convey some personal benefits (social desirability). Secondly, we should consider that the legal circumstances of each participant can modify their types of responses. Thirdly, the participants were subjected to long sentences, thus making it difficult to generalise the results to inmates with shorter sentences. In psychiatric evaluation, we should have included the screening for attention-deficit/hyperactivity disorder due to its high co-morbidity with ASPD [75]. Finally, the study’s results cannot be generalised to ASPD in the general population.

In future research, including assessment tools that externally, longitudinally quantify the individual acts of aggression may prove useful, such as the Modified Overt Aggression Scale. Future studies should additionally include ASPD patients from a general population sample.

## Conclusions

The type of aggression that is associated with ASPD is mainly impulsive in nature. Individuals with emotional dysfunction have a lower probability of exhibiting impulsive aggression and a higher probability of exhibiting premeditated aggression. Although ASPD patients have high levels of impulsivity and a high frequency of SUD, these two variables were not predictors of aggression type.

In order to obtain validated evidence that may guide the treatment of aggression in ASPD patients and translate research concerning aggression to clinical practice in forensic settings, the clear definition of aggression type may benefit the design of clinical trials in ASPD patients [8, 14].

The high prevalence of aggressive acts and co-morbid psychiatric disorders in forensic settings requires a complex and integrated psychiatric assessment that should address the needs of organised forensic psychiatric services in prisons.
